# County-Level Influenza-Attributable Emergency Department Visits and Their Spatial Correlates in the United States: Cross-Sectional Observational Study

**DOI:** 10.2196/82879

**Published:** 2025-12-30

**Authors:** Xucheng (Fred) Huang, Joshua L Warren, Shuqi Lin, Stefanie Ebelt, Howard H Chang

**Affiliations:** 1 Gangarosa Department of Environmental Health Rollins School of Public Health Emory University Atlanta, GA United States; 2 Department of Biostatistics Yale School of Public Health Yale University New Haven, CT United States; 3 Department of Epidemiology Rollins School of Public Health Emory University Atlanta, GA United States; 4 Department of Biostatistics Rollins School of Public Health Emory University Atlanta, GA United States

**Keywords:** infectious diseases, air pollution, environmental epidemiology, environmental disparities, spatial statistics, bayesian statistics, influenza diseases

## Abstract

**Background:**

The burden of seasonal influenza on emergency department (ED) visits is poorly quantified due to case ascertainment and data availability challenges. This study estimates county-level respiratory ED visits attributable to influenza using time-series models and examines spatial heterogeneity in county-level burden in 3 states.

**Objective:**

This study aimed to estimate the county-level respiratory ED visits attributable to influenza using time-series models and examine spatial heterogeneity in county-level burden in 3 states.

**Methods:**

We used daily hospital discharge records to measure community-level influenza activity in California (2005-2018), Georgia (2010-2018), and New York (2005-2018). County-level respiratory ED visit rates attributable to influenza were estimated by quasi-Poisson time-series models, adjusting for temporal trends and environmental factors. Bayesian spatial models were used to assess associations with county-level socioeconomic status, environmental exposures, and chronic health condition prevalence.

**Results:**

Influenza-attributable respiratory ED visit rates per 100,000 population were 226 (95% CI 206-246) in New York, 232 (95% CI 206-259) in California, and 547 (95% CI 506-589) in Georgia. A 10% increase in county-level poverty and uninsured rates was associated with higher influenza burden, increasing influenza-attributable respiratory ED visit rates by 160 (95% credible interval [CrI] 127-196) and 217 (95% CrI 168-265), respectively. Long-term PM_2.5_ (fine particulate matter ≤2.5 µm), humidity, and temperature also exhibited positive associations. Chronic conditions also increased ED visit rates by 1476/100,000 (95% CrI 1167-1778), 588/100,000 (95% CrI 400-747), and 488/100,000 (95% CrI 402-574) per 10% increase in stroke, chronic obstructive pulmonary disease, and diabetes prevalence, respectively. These associations weakened after adjusting for socioeconomic status.

**Conclusions:**

Influenza-attributable respiratory ED visit rates exhibit significant spatial heterogeneity that is associated with county-level socioeconomic factors, environmental exposures, and chronic disease prevalence.

## Introduction

Seasonal influenza is a significant public health concern in the United States, contributing to substantial morbidity, mortality, and health care economic burden. In 2023-24, influenza resulted in an estimated 40 million illnesses, 470,000 hospitalizations, and 28,000 deaths [1]. While influenza-associated hospitalizations and mortality are well documented [2,3], emergency department (ED) visits as a morbidity measure remain underexamined. Quantifying influenza-associated ED burden is important because it can capture both severe cases that lead to hospitalization and mild cases that do not. Also, the percentage of annual ED visits with an influenza test ordered or provided increased from 2.5% in 2013 to 10.9% in 2022, with children aged 0-5 years accounting for the highest testing rates [4]. These rising numbers highlight the growing role of EDs in influenza surveillance and the need for accurate burden estimates to guide program planning.

Despite advances in influenza surveillance, current burden estimates face several limitations. First, many rely on multipliers or prevalence-based models [5], which require extrapolating key parameters from smaller studies. While some incorporate statistical regression models [6], these often overlook key analytical complexities, including temporal autocorrelation, spatial heterogeneity, and environmental confounders. Recent evidence suggests that ambient conditions substantially influence influenza transmission and severity. For example, temperature and relative humidity modulate viral persistence and host susceptibility [7,8], while fine particulate and gaseous pollutants such as PM_2.5_ (fine particulate matter ≤2.5 µm) and NO_2_ (nitrogen dioxide) have been linked to elevated influenza incidence and subtype-specific responses [9,10]. Moreover, broader analyses in temperate regions demonstrate additive effects of air pollution and cold exposure on respiratory morbidity [11]. Hence, studies without accounting for these environmental factors can lead to underestimation and poorer uncertainty quantification.

A further gap in the influenza burden literature is the lack of localized estimates. Most studies use aggregate data at the state [12] and the national level [13], or restrict analyses to specific subpopulations, such as children [14] or veterans [15]. However, growing evidence suggests that other health and environmental factors, such as stroke [16], high BMI [17], air pollution [18], and lower socioeconomic status (SES) [19], can increase vulnerability to respiratory health outcomes. Hence, quantifying influenza disease burden at a finer spatial scale and examining its association with spatial covariates can better identify populations or communities for the development of more effective, targeted interventions.

Motivated by the above research gaps, we aim to estimate county-level seasonal influenza-attributable ED visit rates and associated spatial covariates in 3 US states (California, Georgia, and New York), selected for their geographic diversity and availability of comprehensive hospital data. We first used a time-series model to estimate excess annual seasonal influenza-attributable respiratory ED visits [20]. Next, under a meta-regression framework, we used a spatial Bayesian hierarchical model to examine associations between respiratory ED visit rates and various county-level covariates, including SES, chronic health conditions, and environmental exposures. This study offers a local perspective on the public health impact of seasonal influenza.

## Methods

### Hospital Discharge Records

We obtained patient-level hospital discharge data for 2005-2018 in California and New York, and 2010-2018 in Georgia, from hospital associations and state health departments at the daily level. ED visits included both outpatient visits with discharges directly from the ED and inpatient visits with admissions via the ED, excluding scheduled admissions. Records included information on admission date, age in years, patient’s residential ZIP code, and *ICD* (*International Classification of Diseases)* diagnosis codes. *ICD-9* (*International Classification of Diseases, Ninth Revision*) codes were used before October 1, 2015, after which *ICD-10* (*International Statistical Classification of Diseases, Tenth Revision*) codes were adopted. Both primary and secondary *ICD* diagnosis codes were used to identify cause-specific ED visits for overall respiratory diseases (RESP, *ICD-9* 460-519; *ICD-10* J00-J99) and influenza (FLU, *ICD-9* 487-488; *ICD-10*: J09-J11). This approach captures encounters in which respiratory conditions contributed to the visit but were not coded as the principal diagnosis. For instance, influenza can exacerbate chronic or systemic symptoms. Restricting analyses to primary diagnoses alone could underestimate the true respiratory disease burden, particularly among patients with comorbidities, as shown in previous hospital-based surveillance studies [20-22].

For RESP, FLU, and non-FLU RESP, we created a time series of daily ED visit counts for each county by aggregating ED visits based on the patient’s residential ZIP code that overlapped with these counties. For ZIP codes that overlapped with multiple counties, we assigned ED visits to the county with which the ZIP code had the largest population overlap using the 2010 ZIP Code Tabulation Area (ZCTA) County Relationship File from the US Census Bureau. In cases where population overlap could not be determined (less than 1% of ZIP codes), we assigned the county based on the shortest centroid-to-county distance. There was a total of 279 counties (California n=58, Georgia n=159, and New York n=62).

### Influenza Activity Proxy

The daily influenza activity proxy was constructed from the same hospital discharge dataset of *ICD*-coded influenza ED visits (*ICD-9* 487–488; *ICD-10* J09–J11). We calculated the daily influenza-related ED visit rates per 100,000 population for each county during the influenza season (the Centers for Disease Control and Prevention defines an influenza season typically runs from Morbidity and Mortality Weekly Report (MMWR) week 40 to 18 of the following year [2]), using county-level population data from the US Census Bureau. This variable reflects community-level influenza activity used as the exposure term (*x_t_*) in the quasi-Poisson model described in the Statistical Analysis section. To prevent circularity, influenza-coded visits were removed from the respiratory-disease outcome series when estimating associations. The 2009-2010 influenza A (H_1_N_1_) pandemic season was excluded because it exhibited 2 distinct epidemic peaks and atypical temporal patterns that differed markedly from regular influenza seasons. Including this year could bias the estimation of county-level associations that assume relatively consistent seasonal dynamics. Our analysis thus had 11 influenza seasons analyzed from 2005 to 2018.

### Air Pollution and Meteorology Data

Daily ambient air pollution data for 24-hour average PM_2.5_, 1-hour maximum NO_2_, and 8-hour maximum ozone concentrations from 2005 to 2018 were based on a data product with complete spatial-temporal coverage across the United States at a 12 km × 12 km spatial resolution [23]. These estimates used monitoring data to bias-correct Community Multiscale Air Quality (CMAQ) model simulations that integrate emissions, meteorological conditions, and atmospheric chemistry to improve air quality assessments.

Daily maximum and minimum temperature in degrees Celsius, and water vapor pressure (as a proxy for humidity) in pascals at a 1 km × 1 km spatial resolution were obtained from Daymet [24]. The daily average temperature was calculated as the mean of the daily maximum and minimum.

Air pollution and meteorology data were first aggregated to the ZIP code level, and then up-scaled to the county-level to match the health outcome with population-weighted averaging ZIP code-level exposures. Population size data were obtained from the US Census at the ZCTA level for 2010 to 2020, and we estimated annual populations via linear interpolation of decadal Census data. A 3-day moving average was applied to air pollution and meteorological variables to account for short-term cumulative exposure effects, as the health impacts of environmental exposures are typically not immediate and may manifest over several subsequent days. This approach is consistent with previous studies [18,25,26] on air pollution and meteorological influences on respiratory ED visits that have adopted similar averaging strategies.

### County-Level Covariates

We considered 3 categories of county-level covariates: SES, environmental exposures, and chronic health condition prevalence. SES indicators such as the percent of population living under the poverty line and the percent of uninsured individuals were obtained as 5-year averages (2011-2016) from the American Community Survey. Long-term county-level environmental exposures were obtained by averaging levels of PM_2.5_, NO_2_, O_3_, humidity, and temperature across all influenza seasons (2005-2018). Chronic health conditions were obtained from the 2022 Behavioral Risk Factor Surveillance System, including the prevalence rates of individuals with disability, and other major chronic diseases such as stroke, diabetes, and high blood pressure. Table S1 in Multimedia Appendix 1 summarizes the data sources, variable descriptions, and time units for all covariates.

### Statistical Analysis

#### Stage 1: Estimating County-Level Disease Burden

We conducted excess morbidity modeling based on a previously developed model [20] to estimate the total number of respiratory ED visits attributable to influenza. Specifically, the model estimated the excess respiratory visits associated with influenza activity that were not diagnosed as influenza, which were then combined with *ICD*-coded influenza ED visits to obtain the total influenza-attributable burden. First [20], an overdispersed Poisson log-linear model was used to estimate county-level associations between the daily influenza activity proxy and daily respiratory ED visits, for which visits with influenza listed as a primary or secondary cause were removed to avoid circularity. The analysis was restricted to days within each influenza season. For each county *j*=1,2,…, *m_i_* within state *i* (*i=*1 for California, 2 for Georgia, 3 for New York), where *m_i_* is the number of counties in state *i*, let *Y_ijt_* denote the number of RESP ED visit count (excluding influenza-coded cases) on day *t* in county *j* of state *i*. For simplicity, we drop the county and state subscripts (*i, j*) in the equations below, as the model was applied identically to each county-state combination. We assume *Y_t_* follows a quasi-Poisson distribution with mean λ*_t_* such that



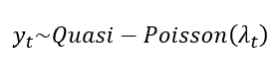





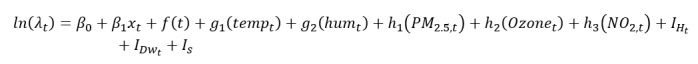



(1)

where (1) *x_t_* is influenza ED visit rate per 100,000 on day *t* (ie, the influenza activity proxy), (2) *f(t)* is a temporal smoother for seasonal and long-term trends modeled using natural cubic splines with 4 degrees of freedom per influenza season, (3) *g_1_(temp_t_)* and *g_2_(hum_t_)* are nonlinear effects of 3-day moving average temperature and humidity using natural cubic splines with 6 degrees of freedom, (4) *h_1_(*PM_2.5_*_,t_)*, *h_2_(Ozone_t_)* and *h_3_(*NO_2_*_,t_)* represent linear effects of 3-day moving average air pollution levels; and (5) *I_Ht_*, *I_DWt_*, and *I_s_* are indicators for national holidays, day of week, and influenza season. For meteorological variables, 6 degrees of freedom were applied to capture U-shaped associations [25]. To address residual autocorrelation, we utilized the Newey-West estimator [27] with a maximum lag of 2 to calculate the SEs of each model coefficient.

For each county *j* in state *i*, we calculated the average annual ED visits attributable to influenza by first summing, over the entire influenza season (*S*) for state *i*, the difference between the predicted counts with the observed flu proxy rate and the counterfactual expected counts assuming there is no influenza activity (ie, setting covariates *x_t_* to zero). This total was then divided by the number of flu seasons, *N_s_.* The computation is shown in equation (2):



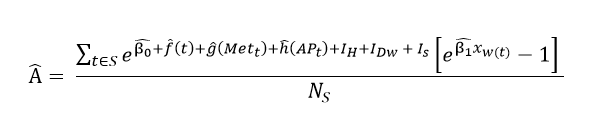



(2)

Where Â can be interpreted as the expected excess number of influenza-attributable ED visits per influenza season that were not ICD-coded. The *ĝ*(*Met_t_*) denotes the combined effects of temperature and humidity, while the *ĥ*(*AP_t_*) denotes the aggregate impact of PM_2.5_, ozone, and NO_2_.

The resulting estimate for each county *j* in state *i* is denoted as Â*_ij_*. The delta method was applied to obtain standard errors and construct 95% CIs for the influenza-attributable respiratory ED visits, Â*_ij_*. To determine the total influenza attributable counts, we added back the average number of *ICD*-coded influenza ED visits. Finally, we calculated the annual total respiratory ED visits rates attributable to influenza per 100,000 population, 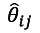, using the formula below:



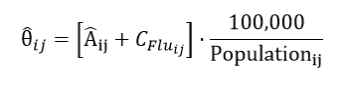



(3)

where 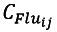 is the annual *ICD*-coded influenza ED counts, calculated by averaging the total flu-coded counts across the entire influenza season *S_i_*, and *Population_ij_* denotes the catchment population for county *j* in state *i*.

To summarize the overall burden at the state level, we aggregated county-specific influenza-attributable respiratory ED visit rates 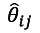 and their standard errors using an inverse-variance weighted approach. The resulting state-level estimates represent the overall influenza-attributable respiratory burden for each state. We further combined the 3 state-specific estimates using a random-effects meta-analysis to obtain a pooled estimate reflecting the overall burden across all study states.

#### Stage 2: Bayesian Spatial Hierarchical Modeling

We used spatial meta-regression [28] to examine associations between county-level rates of respiratory ED visits attributable to influenza and spatial covariates while accounting for spatial autocorrelation. The true county-level burden rate *θ_ij_* is modeled as:



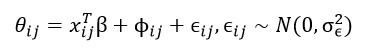



(4)

Where 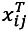 is a vector of county-level covariates with corresponding regression coefficients. 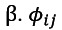 is a random spatial effect that captures residual spatially dependent heterogeneity among counties in each state. We modeled 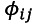 with a proper conditional autoregressive model specification [29], independent across states, where spatial adjacency is defined as two counties that share a border. Finally, 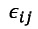 is an independent random error term with a state-specific variance parameter that accounts for additional between-county variation. The full specification of the hierarchical spatial model is provided in Multimedia Appendix 2*.*

**Figure 1:**
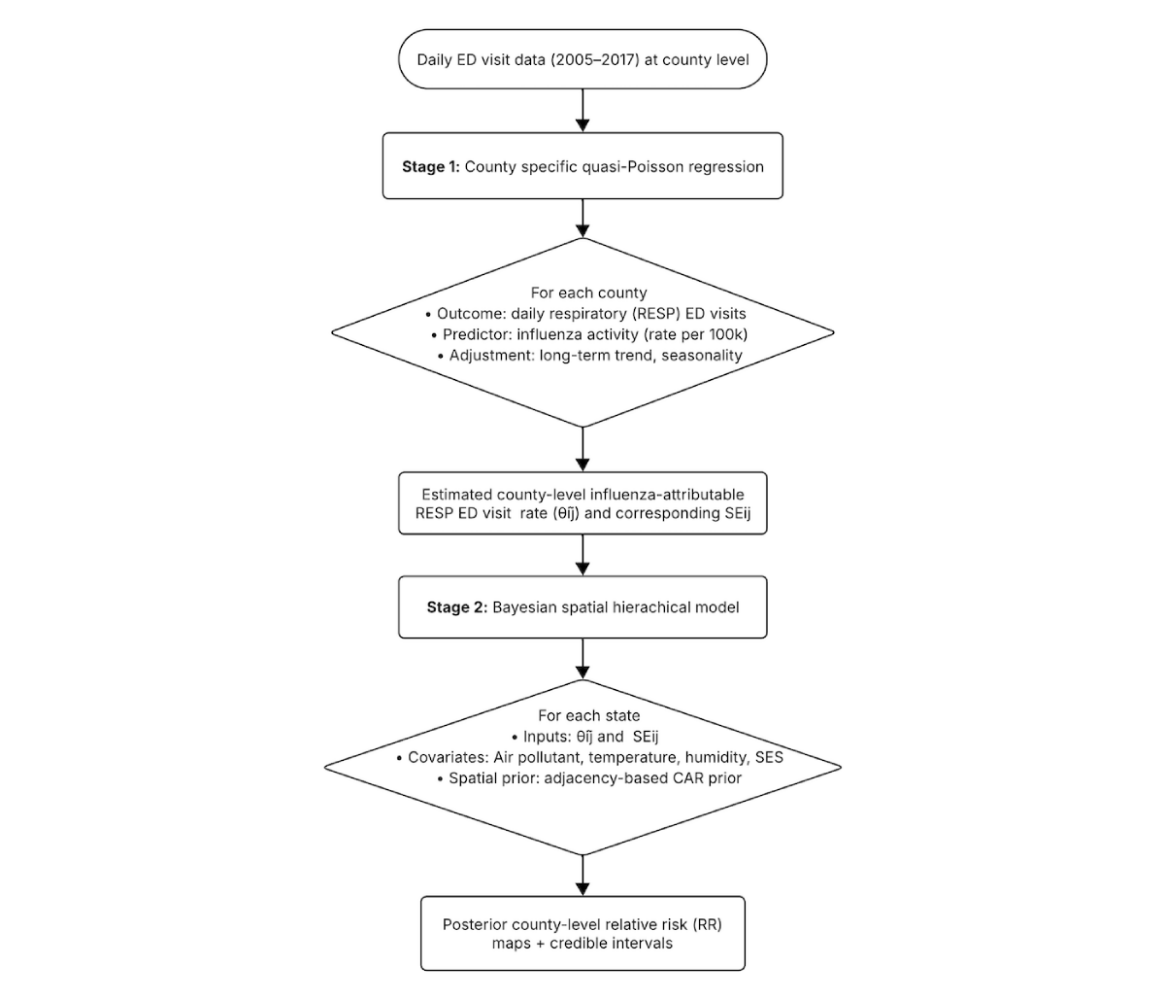
Overview of the 2-stage modeling framework for estimating county-level influenza-attributable respiratory emergency department (ED) visit rates and examining their spatial associations with covariates. RESP: respiratory disease; CAR: conditional autoregressive model.

In this stage, we assumed linear relationships between long-term county-level covariates and influenza-attributable ED visit rates. We first assessed univariate associations for each spatial covariate. To account for potential nonlinear population effects, in a secondary analysis, we included the logarithm of the population as a cubic spline with 3 degrees of freedom. Additionally, we examined the associations between environmental exposures or chronic health outcomes after adjusting for SES (poverty and uninsured rates) in multivariable models. Figure 1 illustrates the workflow of the 2-stage modeling framework for estimating county-level influenza-attributable respiratory ED visit rates.

We conducted a sensitivity analysis restricted to a high-severity influenza season (2017-2018) to evaluate the robustness of results. To further examine the stability of temperature and humidity effects in stage 2, we refitted the Stage-2 model as a secondary analysis with dummy indicators for mean temperature and humidity levels, categorized into 3 groups (≤33rd, 34th-66th, and ≥67th percentiles) and 3 ranges capturing extreme conditions (≤10th, 19th-90th, and ≥90th percentiles), separately. Data compilations were conducted in SAS Statistical Software (version 9.4; SAS Institute), and statistical analyses were conducted in R software (version 4.0.3; R Foundation for Statistical Computing).

### Ethical Considerations

This study used secondary, deidentified hospital discharge data obtained under active data use agreements with state health departments and hospital associations. The dataset spans multiple US states and years (2005-2018) and includes both outpatient and inpatient discharge records. All data were provided under institutional agreements for environmental health research and stored on secure university servers. The study protocol was reviewed and approved by the Emory University Institutional Review Board (STUDY00006348, 2024P007407). Informed consent was not required because the data were fully deidentified before access and analysis. Only ZIP code–level admission and diagnostic information were available, and all analyses were performed using aggregated county-level data. All datasets were anonymized before analysis, and no attempt was made to reidentify individuals. No participant compensation was applicable for this study.

## Results

The study included 61.81 million RESP ED visits, of which 1.52 million (2.5%) were coded as influenza during the influenza season. Table 1 summarizes the ED visit counts by state. California accounted for approximately 33.17 million RESP ED visits (of which 0.78 million, 2.4%, were coded as influenza), Georgia had 7.48 million RESP ED visits (0.31 million, 4.1%, influenza-coded), and New York for 21.16 million RESP ED visits (0.43 million, 2.0%, influenza-coded).

Table 2 provides summary statistics for the spatial covariates across 279 counties. Poverty and uninsured rates varied widely (median 13.5%, IQR 9.65% to 18.3% and median 14.1%, IQR 8.95% to 16.95%, respectively). Environmental exposures also showed notable spatial variability during influenza seasons: temperature had a median of 12.63 °C (IQR 7.78 to 14.32 °C), humidity 7.09 g/kg (IQR 5.01 to 8.06 g/kg), PM2.5 7.75 µg/m³ (IQR 7.31 to 8.17 µg/m³), and NO2 8.19 ppb (IQR 5.77 to 13.27 ppb). Among chronic conditions, high blood pressure had the greatest variability (median 33.70%, IQR 28.85% to 39.05%), while asthma had the least (median 10.40%, IQR 10.40% to 11.30%).

**Figure 2:**
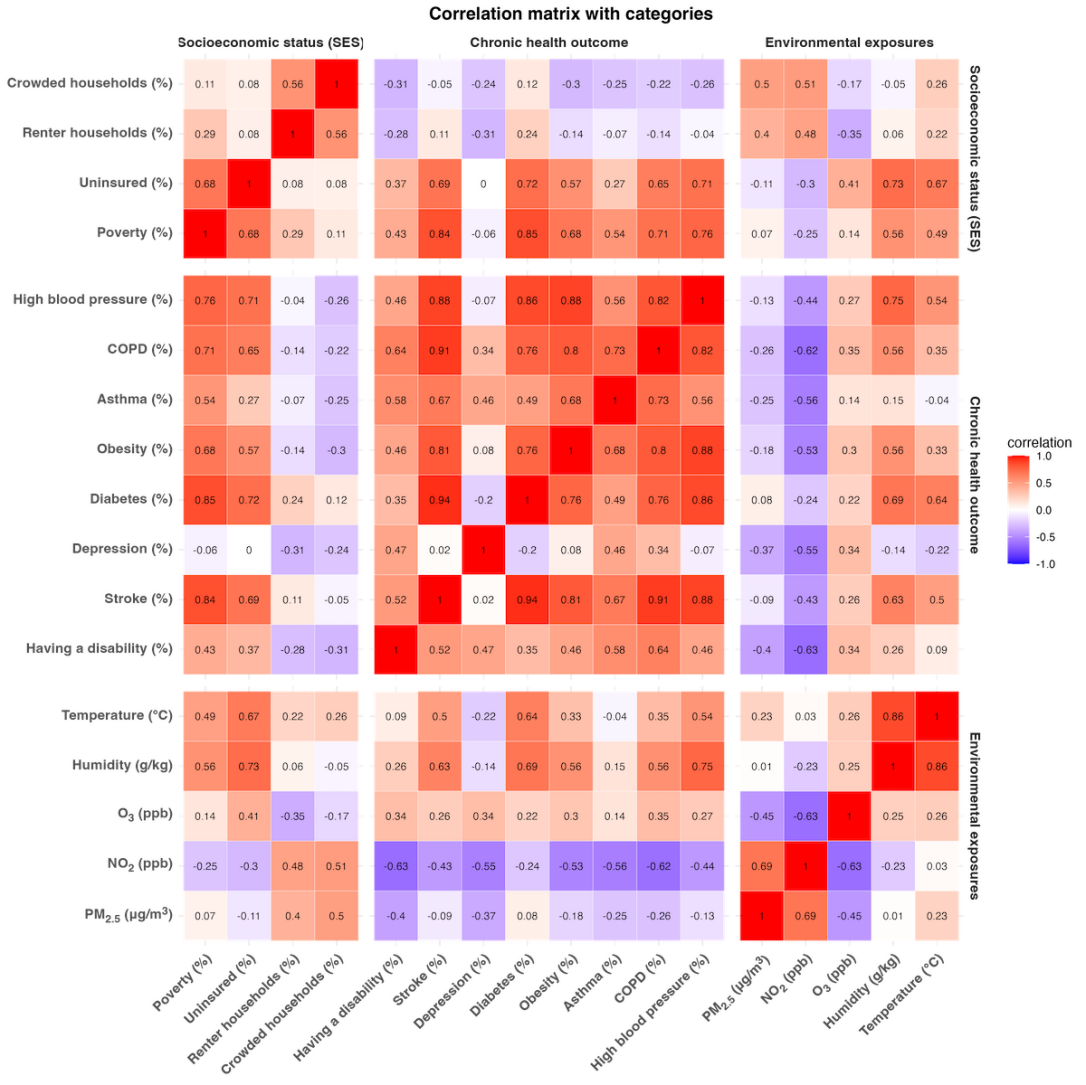
Pairwise Pearson correlations heatmap of spatial covariates across socioeconomic status (SES), chronic health outcomes, and environmental exposures (Counties, n=279). Pct_pov (%): % living below poverty line; pct_uninsured: % without health insurance; pct_renter: % renter households (lower homeownership); pct_crowd: % crowded households (multiple occupants per room); pct_a_disability: % with at least one disability among hearing, vision, cognitive, ambulatory, self-care, or independent living difficulty; BPHIGH: high blood pressure (hypertension); MeanHumid: mean humidity; MeanTemp: mean temperature; COPD: chronic obstructive pulmonary disease; NO2: nitrogen dioxide; O3: ozone; PM2.5: fine particulate matter ≤2.5 µm.

Figure 2 presents pairwise correlations among county-level spatial covariates. Strong positive correlations were observed within SES indicators (eg, poverty and uninsured rates, *r*=0.68) and among chronic health outcomes, with high blood pressure strongly correlated with obesity, stroke, diabetes, and chronic obstructive pulmonary disease (COPD; *r*=0.82 to 0.88). Environmental exposures exhibited moderate correlations with SES and health outcomes (r=–0.63 to 0.75). Within environmental exposures, strong positive correlations were observed between PM_2.5_ and NO_2_ (*r*=0.69) and between humidity and temperature (*r*=0.86).

State-level estimates of influenza-attributable respiratory ED visit rates per 100,000 population and a pooled estimate across the 3 states are presented in Table S2 in Multimedia Appendix 1*.* Georgia had the highest burden (547/100,000; 95% CI 506-589), followed by California (232/100,000; 95% CI 206-259) and New York (226/100,000; 95% CI 206-245). *ICD*-coded influenza ED visits represented 54% (California), 75% (Georgia), and 62% (New York) of the total estimated influenza-attributable ED visits. Using a random-effects meta-analysis, the pooled burden estimate across the 3 states was 410/100,000 (95% CI 379, 440).

**Figure 3:**
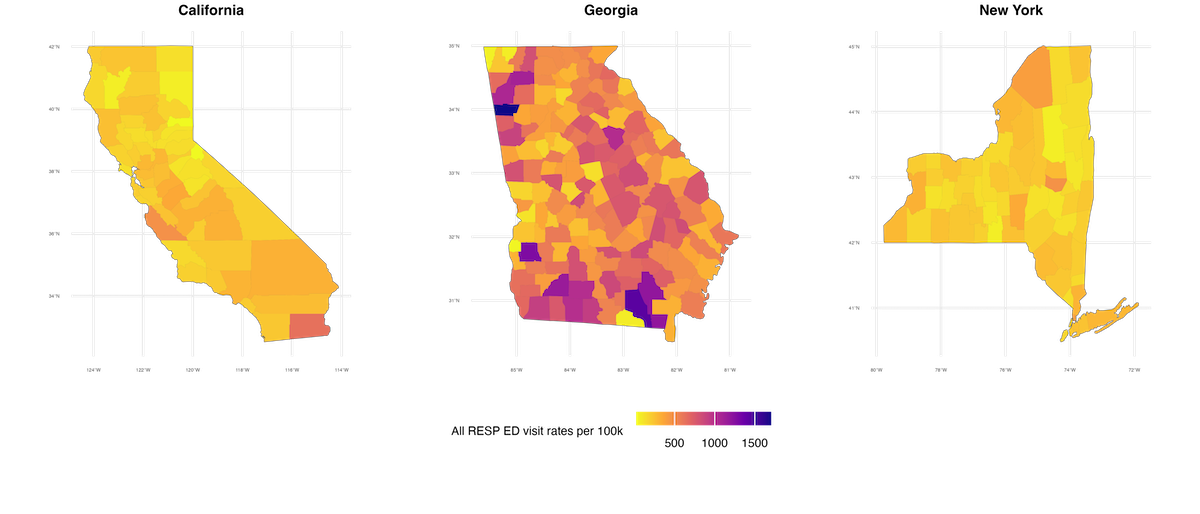
Heatmap of annual estimated influenza-attributable respiratory emergency department visit rates per 100,000 at the county level in California, Georgia, and New York from 2005-2006 to 2017-2018. Rates are averaged across all influenza seasons during the study period. RESP: respiratory disease; ED: emergency department.

Figure 3 shows the estimated county-level influenza-attributable respiratory ED visit rates from the stage 1 model. These were calculated by summing seasonal ED visits (see Figure S1 in Multimedia Appendix 1 for visual distributions and Multimedia Appendix 3 for numerical data) and then dividing by the number of influenza seasons for each county. Georgia showed the highest spatial variability, with several counties in the southern and central regions having annual influenza-attributable respiratory ED visit rates exceeding 1500 per 100,000. In contrast, California and New York had lower rates. Most counties in California had ED visit rates below 1000 per 100,000 in California, except for a few hotspots in the southern region. New York displayed a more consistent pattern, with most counties having moderate rates between 500 and 1000 per 100,000. The Moran *I* statistics (see Table S3 in Multimedia Appendix 1) confirm the spatial heterogeneity across the study regions. Maps of influenza-attributable respiratory ED visit rates per 100,000 for each season are given in Figure S1 in Multimedia Appendix 1*.* Numerical values of all estimated rates with 95% CI by state and influenza season are in Multimedia Appendix 1*.*

Table 3 summarizes the spatial meta-regression results. Results from Model 1 (univariate analyses) identified positive associations between SES, environmental exposures, and chronic health factors with influenza-attributable respiratory ED visit rates. A 10% increase in higher percentages of poverty and uninsured populations was strongly associated with increased ED visit rates, with estimated increases of 160/100,000 (95% credible interval [CrI] 127-196) and 217/100,000 (95% CrI 168-265), respectively. For environmental exposures, notable positive associations were observed for higher humidity, with an increase of 222/100,000 (95% CrI 183-260) ED visit rates per IQR increase, and temperature, with an increase of 71/100,000 (95% CrI 42-108) per IQR increase. In contrast, PM_2.5_ showed a smaller association, with an increase of 14/100,000 (95% CrI 6-22) per IQR increase. NO_2_ exhibited a comparable effect size of 14 per 100,000 (95% CrI –0.1 to 27), and although the lower bound slightly crosses zero, the association remains noteworthy. Ozone had negligible associations with influenza-attributable respiratory ED visit rates.

Chronic health outcomes also showed strong positive associations with influenza-attributable RESP ED visit rates. The percentage of stroke had the largest association, with an increase of 1476/100,000 (95% CrI 1167-1778) per 10% increase in county-level prevalence, followed by diabetes (488/100,000, 95% CrI: 402-574) and COPD (588/100,000, 95% CrI 400-747). Other chronic health factors, such as obesity, asthma, and high blood pressure, were also positively associated with increases in influenza-attributable respiratory ED visit rates.

These results, also presented in Table 3, are robust to adjustment for the prevalence of poverty (Model 2), uninsured (Model 3), or both (Model 4), but with attenuated associations. The poverty and uninsured rates remained positively associated with ED visit rates across all models, or when they were both included in the model (see Table S4 in Multimedia Appendix 1). Environmental exposures, including humidity and temperature, continued to exhibit positive associations, while associations with air pollution became null after SES adjustment. Chronic health outcomes, including stroke, diabetes, and COPD, continued to show strong positive associations, with stroke remaining the strongest association with influenza-attributable respiratory ED visit rates, followed by asthma, COPD, high blood pressure, and obesity.

After adjusting for nonlinear population effects, most associations remained consistent, with slightly stronger increasing trends observed (see Table S5 in Multimedia Appendix 1). The prevalence of having a disability, which showed no significant association in the previous univariate model, became positively associated with ED visit rates (86/100,000, 95% CI 32-143). Ozone exposure was also positively associated with ED visit rates after adjustment for population, while associations for PM_2.5_ and NO_2_ became negatively associated after SES and population adjustment. We also fitted models with population as the only covariate (see Figure S2 in Multimedia Appendix 1). There is a nonlinear association between log population and influenza-attributable respiratory ED visit rates, with sharper increases at lower population levels and a flattening association as population size increases.

The results of primary sensitivity analysis (see Table S6 in Multimedia Appendix 1) showed that associations between spatial covariates and influenza-attributable respiratory ED visit rates remained in the same directions as the primary analyses, though with larger effect estimates. For instance, a 10% increase in stroke prevalence during the 2017-2018 influenza season was associated with a 2614/100,000 (95% CI 2143-3075) increase in influenza-attributable respiratory ED visit rates.

**Table 1:** Summary of emergency department visit counts for ICD-coded influenza and respiratory disease across California (2005-2018), Georgia (2010-2018), and New York (2005-2018).

	RESP^a^ ED^b^ visits, n	Flu ED visits, n (%)	Nonflu RESP, n (%)	Years covered	Number of counties
California	33,172,708	781,310 (2.4)	32,391,398 (97.6)	2005-2018	58
Georgia	7,481,121	306,234 (4.1)	7,174,887 (95.9)	2010-2018	159
New York	21,160,209	432,495 (2)	20,727,714 (98)	2005-2018	62
Total	61,814,038	1,520,039 (2.5)	60,293,999 (97.5)	—^c^	279

^a^RESP: respiratory disease.

^b^ED: emergency department.

^c^Not available.

**Table 2:** Summary statistics of spatial covariates at the county level across California, Georgia, and New York.

Covariates			Median (IQR)		Range		Mean (SD)
**Socioeconomic status (SES)**							
	Poverty (%)	13.5 (9.65 to 18.3)		2.70 to 38.40		14.26 (6.14)	
	Uninsured (%)	14.1 (8.95 to 16.95)		4.40 to 32.80		13.63 (5.09)	
	Renter (%)	32.15 (26.92 to 38.93)		14.85 to 80.93		33.56 (9.98)	
	Crowding (%)	2.20 (1.52 to 3.61)		0.16 to 12.82		3.05 (2.37)	
**Environmental exposures**							
	PM_2.5_ (μg/m^3^)	7.75 (7.31 to 8.17)		3.89 to 12.68		7.84 (1.17)	
	NO_2_ (ppb)	8.19 (5.77 to 13.27)		1.07 to 43.15		10.52 (7.09)	
	Ozone (ppb)	36.66 (35.25 to 37.20)		23.37 to 43.70		36.22 (2.33)	
	Humidity (g/kg)	7.09 (5.01 to 8.06)		2.86 to 10.66		6.72 (1.74)	
	Temperature (°C)	12.63 (7.78 to 14.32)		–10.80 to 17.18		10.67 (5.06)	
**Chronic health outcome**							
	Disability (%)	15.1 (12.70 to 17.70)		7.00 to 27.00		15.2 (3.77)	
	Stroke (%)	3.40 (3.00 to 4.10)		2.10 to 6.30		3.56 (0.77)	
	Depression (%)	22.7 (21.30 to 23.80)		14.8 to 26.9		22.42 (2.05)	
	Diabetes (%)	11.10 (9.40 to 13.05)		7.10 to 18.10		11.33 (2.25)	
	Obesity (%)	37.10 (33.59 to 41.60)		17.70 to 48.60		37.70 (5.71)	
	Asthma (%)	10.90 (10.40 to 11.30)		8.50 to 13.10		10.80 (0.73)	
	COPD^a^ (%)	7.30 (6.10 to 8.55)		3.20 to 12.10		7.32 (1.72)	
	BPHigh^b^ (%)	33.70 (28.85 to 39.05)		21.60 to 47.8		34.03 (5.82)	

^a^COPD: chronic obstructive pulmonary disease.

^b^BPHigh: high blood pressure (hypertension).

**Table 3:** Results from spatial meta-regression models examining associations between county-level covariates and annual influenza-attributable respiratory emergency department (ED) visit rates per 100,000 population.

Covariates			Model 1^a^		Model 2^b^		Model 3^c^		Model 4^d^
**Socioeconomic status (SES)**									
	Poverty (× 10%^e^)	160 (127 to 196)		—^f^		121 (84 to 159)		—	
	Uninsured (× 10%)	217 (168 to 265)		115 (66 to 167)		—		—	
**Environmental exposures**									
	PM_2.5_ (× IQR^g^ μg/m^3^)	14 (6 to 22)		2 (–7 to 10)		9 (–1 to 18)		2 (–8 to 11)	
	NO_2_ (× IQR ppb)	14 (–0.1 to 27)		2 (–11 to 15)		3 (–13 to 18)		0 (–14 to 14)	
	Ozone (× IQR ppb)	6 (–2 to 13)		4 (–3 to 12)		–4 (–12 to 4)		–1 (–9 to 7)	
	Humidity (× IQR g/kg)	222 (183 to 260)		148 (109 to 187)		151 (104 to 197)		130 (87 to 174)	
	Temperature (× IQR °C)	71 (42 to 108)		35 (14 to 58)		29 (2 to 58)		20 (–3 to 44)	
**Chronic health outcome**									
	Having a disability (× 10%)	26 (–23 to 79)		–45 (–86 to –1)		13 (–38 to 64)		–35 (–81 to 12)	
	Stroke (× 10%)	1476 (1167 to 1778)		699 (243 to 1164)		994 (653 to 1336)		467 (6 to 933)	
	Depression (× 10%)	–19 (–72 to 35)		–57 (–106 to –1)		–238 (–80 to 33)		–48 (–101 to 5)	
	Diabetes (× 10%)	488 (402 to 574)		383 (244 to 521)		410 (301 to 517)		297 (138 to 456)	
	Obesity (× 10%)	124 (89 to 163)		48 (16 to 82)		88 (58 to 119)		56 (25 to 89)	
	Asthma (× 10%)	196 (57 to 340)		–45 (–185 to 95)		149 (6 to 294)		–1 (–147 to 145)	
	COPD^h^ (× 10%)	588 (400 to 747)		157 (1 to 313)		308 (174 to 445)		145 (8 to 289)	
	BPHigh^i^ (× 10%)	217 (183 to 250)		152 (108 to 194)		169 (131 to 209)		139 (95 to 184)	

^a^Model 1 includes univariate associations.

^b^Model 2 adjusts for poverty rates.

^c^Model 3 adjusts for uninsured rates.

^d^Model 4 adjusts for both poverty and uninsured rates (coefficients of the two socioeconomic statuses are in Table S4 in Multimedia Appendix 1). Posterior mean point estimates and 95% quantile-based credible intervals are presented.

^e^Covariates expressed as “× 10%” were scaled such that coefficients represent the rate difference associated with a 10-percentage-point increase in the corresponding percentage variable.

^f^Not available.

^g^Environmental exposure covariates expressed as “× IQR” were scaled to the IQR; estimates represent the change in ED visit rates associated with an increase equal to one IQR of the exposure.

^h^COPD: chronic obstructive pulmonary disease.

^i^BPHigh: high blood pressure (hypertension).

## Discussion

### Principal Findings

We analyzed influenza-attributable respiratory ED visits from 2005-2006 to 2017-2018 across 3 US states and identified substantial geographic heterogeneity associated with socioeconomic, environmental, and chronic disease factors. By leveraging statewide hospital discharge data spanning more than a decade, our study provided a comprehensive assessment of influenza-related respiratory disease burden. The meta-regression framework within the Bayesian spatial modeling approach enabled the quantification of geographic variability and offered a deeper understanding of county-level influenza-attributable respiratory visit burdens.

Substantial regional heterogeneity in ED burden was observed, with Georgia exhibiting rates over twice those of California and New York. This may reflect differences in SES, health care access, population health, and health-seeking behaviors. Georgia also had the highest proportion of all respiratory ED visits coded as influenza (306,234/7,481,121, 4.1%) and higher baseline rates of poverty and chronic health conditions.

Meta-regression analysis revealed that county-level higher poverty rates and lower insurance rates were associated with higher rates of respiratory ED visits attributable to influenza. This aligns with previous research indicating that social determinants of health play a crucial role in chronic respiratory disease disparities [30], with lower SES being associated with severe respiratory conditions and more ED visits.

For environmental exposures, higher levels of PM_2.5_, humidity, and temperature were associated with increased influenza-attributable respiratory ED visit rates in univariate models. Among these factors, humidity exhibited the strongest association, followed by temperature. However, these findings contrast with previous research [31,32], suggesting peak influenza transmission occurs during colder, drier conditions. One explanation is that ED visits capture both new infections and exacerbations of existing conditions, whereas previous studies have primarily focused on influenza virus transmission alone. Moreover, elevated humidity, particularly during the influenza season, which largely overlaps with the winter period in our study, may worsen respiratory illness through mechanisms such as indoor dampness and mold [33]. Even modest increases in wintertime temperature may lead to more outdoor activity or allergen exposure, triggering respiratory events [34]. Additionally, previous studies have generally examined temperature and humidity across the full annual range, whereas our analysis focused on modest rises within the influenza season. This distinction may explain why our findings differ: even though influenza transmission is typically higher in winter compared with warmer periods of the year, higher temperature and humidity within the influenza season appear to have positive associations with influenza-attributable respiratory outcomes. The secondary sensitivity analysis’s results (see Table S7 in Multimedia Appendix 1) support these observed positive associations by showing that the positive associations between temperature, humidity, and respiratory ED visits remained consistent across stratified levels. Both higher temperature and humidity within the influenza season and extremely cold conditions were associated with increased ED visit rates.

A small but positive association between PM_2.5_ levels and influenza-attributable respiratory ED visit rates was observed in the univariate models. This finding is consistent with previous research [26] showing a positive link between ambient PM_2.5_ and respiratory diseases and respiratory-related ED visits. After adjusting for nonlinear population effects, we observed a statistically significant positive association between ozone and influenza-attributable respiratory ED visit rates, suggesting that ozone may contribute to respiratory burden when factors associated with population size are accounted for. This finding is supported by previous epidemiological studies that link ambient ozone exposures to airway inflammation and increased hospital admissions for asthma and other respiratory conditions [35]. However, the association between PM_2.5_ and respiratory ED visit rates became significantly negative after adjusting for both the nonlinear population size and 2 SES factors. We did not find other studies reporting a protective effect between PM_2.5_ and health outcomes. This unexpected result may be due to the positive correlations between population size and PM_2.5,_ and the negative association of PM_2.5_ may be due to model misspecification of the population association. Further investigation is needed to elucidate these associations.

Our findings demonstrated strong associations between the prevalence of county-level chronic health conditions and influenza-attributable respiratory ED visit rates. Among these conditions, stroke exhibited the strongest estimated association across all models. This may be attributed to the long-term effects of cerebrovascular disease, including compromised immune function [36], increased susceptibility to infection, and difficulties in clearing respiratory pathogens due to weakened muscular function [37]. Previous studies have shown that stroke survivors are at an elevated risk of pneumonia and other respiratory infections [38].

Diabetes and COPD rates also showed the strongest associations. These findings align with existing evidence that diabetes and COPD significantly contribute to respiratory health complications. Diabetes impairs immune response, increasing susceptibility to infections, including influenza and pneumonia [39], while COPD is characterized by chronic lung inflammation and reduced pulmonary function [40], making individuals more prone to severe respiratory infections and exacerbations requiring emergency care.

Adjusting for poverty and uninsured rates attenuated most associations, suggesting that part of the association between county-level chronic disease and influenza-attributable respiratory ED visits may reflect underlying socioeconomic disadvantage. The effects of poverty and uninsured prevalence remained similar and increased in some 2-SES adjusted models. If chronic conditions are a mediating factor between SES and influenza-attributable respiratory ED visits, we would expect SES effects to decrease when controlling for health status. The observed stronger association points to **an independent and possibly more direct role** of SES in shaping emergency care use. Although individuals with lower SES often face barriers to seeking health care, they also experience higher disease burden and tend to rely on EDs as their primary point of care due to limited access to preventive services and insurance coverage [41,42]. This reliance on EDs as safety-net facilities may explain why poverty and uninsured prevalence remain positively associated with respiratory ED visit rates, even after accounting for chronic disease burden [43]. The persistent SES effect likely reflects both greater underlying need and structural inequities in healthcare access and continuity.

Our findings have important implications for public health practice and intervention. County-level identification of areas with elevated influenza-attributable respiratory ED visit rates can help local health departments prioritize limited resources, such as staffing, testing capacity, and antiviral distribution, during peak influenza seasons. In addition, integrating spatial patterns of socioeconomic disadvantage and chronic-disease burden can inform more equitable vaccination outreach and community-based prevention programs. For example, counties with persistently high respiratory ED burden and high poverty or uninsured rates may benefit from tailored vaccination drives, mobile clinics, or public health messaging to enhance early care-seeking behaviors. These results demonstrate how spatially resolved influenza burden estimates can support targeted mitigation and preparedness strategies at the community level.

This study has several limitations. First, our reliance on *ICD*-coded influenza ED visit data as a proxy for influenza burden may still be subject to misclassification bias, given that coding practices vary across hospitals and over time. However, we conducted a validation analysis by comparing *ICD*-coded influenza hospitalizations from our datasets to laboratory-confirmed influenza cases reported by the US Influenza Hospitalization Surveillance Network (FluSurv-NET) surveillance system [44]. In counties with overlapping data, we aligned weekly counts from both sources using the MMWR-week framework. Pearson correlation analysis (see Figure S3 in Multimedia Appendix 1) demonstrated good agreement between *ICD*-coded hospitalizations and FluSurv-NET data.

Second, our analysis areas may not fully generalize to other regions with different health care access, demographics, or environmental exposures. Third, although we considered key environmental and socioeconomic variables, important factors such as vaccination status, healthcare-seeking behavior, and medical history were unavailable at the county level.

### Conclusion

This study provides a county-level assessment of influenza-attributable respiratory ED visits across California, Georgia, and New York. Our findings highlight the importance of SES, environmental exposures, and chronic health conditions as key spatial predictors of local influenza-related respiratory ED visit rates. Counties with higher poverty and uninsured rates, greater humidity and temperature, and elevated prevalence of chronic conditions such as stroke, COPD, and others, were strongly linked to higher influenza-attributable respiratory ED visits. These findings highlight the need for targeted interventions, such as improving health care access, mitigating environmental risks, and managing chronic diseases, to reduce influenza severity and demonstrate the value of incorporating spatial data into public health planning.

## Appendix

Multimedia Appendix 1 Data sources, variable descriptions, covariate distributions, influenza-attributable ED visit estimates, spatial autocorrelation statistics, and spatial meta-regression results.

Multimedia Appendix 2 The detailed modeling process of the Bayesian hierarchical modeling as described in the Stage 2 statistical modeling section.

Multimedia Appendix 3 The numeric values for county-level influenza-attributable respiratory disease emergency department visit rates for each season.

## Abbreviations

CMAQ: Community Multiscale Air Quality
COPD: chronic obstructive pulmonary disease
CrI: credible interval
ED: emergency department
FluSurv-NET: Influenza Hospitalization Surveillance Network
ICD: International Classification of Diseases
ICD-9: International Classification of Diseases, Ninth Revision
ICD-10: International Statistical Classification of Diseases, Tenth Revision
MMWR: Morbidity and Mortality Weekly Report
PM₂.₅: fine particulate matter ≤2.5 µm
RESP: respiratory disease
SES: socioeconomic status
ZCTA: ZIP Code Tabulation Area

## Data Availability

The hospital discharge data used in this study are not publicly available due to restrictions under existing Data Use Agreements. Interested parties can directly request these records from state-specific data custodians. County-level effect modifiers used in the analyses were derived from publicly available sources. Additional information may be obtained from the corresponding author upon reasonable request.
