# Supplementary material for: County-Level Influenza-Attributable Emergency Department Visits and Their Spatial Correlates in the United States: Cross-Sectional Observational Study
Source: JMIR Public Health Surveill. 2025 Dec 30;11:e82879. doi: 10.2196/82879 (PMC12800733; doi:10.2196/82879)

**Table S1.** Summary of variables and data sources for the analysis of influenza-attributable respiratory emergency department (ED) visits in California (CA), Georgia (GA), and New York NY).

| **Category** | **Variable** | **Description** | **Source** | **Years Covered** |
| --- | --- | --- | --- | --- |
| **Influenza Activity Proxy** | FLU ED Visits | Rate of ED visits (inpatient and outpatient) with an ICD code for influenza (per 100,000 population) | Hospital Discharge Records | 2005–2018 (CA, NY), 2010–2018 (GA) |
| **Air Pollution** | PM_2.5_ | 24-hour average fine particulate matter (µg/m³) | CMAQ Model | 2005–2018 |
|  | NO_2_ | 1-hour maximum nitrogen dioxide (ppb) | CMAQ Model | 2005–2018 |
|  | Ozone | 8-hour maximum ozone (ppb) | CMAQ Model | 2005–2018 |
| **Meteorology** | Temperature | Daily maximum and minimum temperature (°C) | Daymet | 2005–2018 |
|  | Humidity | Vapor pressure as a proxy for humidity (g/kg) | Daymet | 2005–2018 |
| **Socioeconomic Status (SES)** | Pct_pov | Percentage of population below poverty line (%) | ACS | 2011–2016 (5-year average) |
|  | Pct_uninsured | Percentage of uninsured individuals (%) | ACS | 2011–2016 (5-year average) |
|  | Pct_renter | Percentage of renter households (%) | ACS | 2011–2016 (5-year average) |
|  | Pct_crowd | Percentage of overcrowded households (>1 occupant per room) (%) | ACS | 2011–2016 (5-year average) |
| **Chronic Health** | Pct_a_disability | Prevalence of having disability (%) | ACS | 2011–2016 (5-year average) |
|  | Stroke | Prevalence of stroke (%) | BRFSS | 2022 |
|  | Depression | Prevalence of depression (%) | BRFSS | 2022 |
|  | Diabetes | Prevalence of diabetes (%) | BRFSS | 2022 |
|  | Obesity | Prevalence of obesity (%) | BRFSS | 2022 |
|  | Asthma | Prevalence of asthma (%) | BRFSS | 2022 |
|  | COPD | Prevalence of chronic obstructive pulmonary disease (%) | BRFSS | 2022 |
|  | BPHigh | Prevalence of high blood pressure (%) | BRFSS | 2022 |

**Hospital Discharge Records:** Patient-level discharge data obtained from hospital associations and state health departments in California, Georgia, and New York (2005–2018 for CA and NY, 2010–2018 for GA).

**CMAQ (Community Multi-scale Air Quality):** Modeled air pollution estimates incorporating ambient monitoring measurements and meteorological simulations. Data provided at a 12 km × 12 km spatial resolution.

**Daymet:** Daily gridded meteorological data including temperature and vapor pressure, available at a 1 km × 1 km spatial resolution.

**ACS (American Community Survey):** Five-year estimates (2011–2016) of socioeconomic and other indicators provided by the U.S. Census Bureau.

**BRFSS (Behavioral Risk Factor Surveillance System):** County-level prevalence rates of chronic health conditions from the CDC.

*Note: Socioeconomic Status (SES) covariates include:

- **Pct_pov (%):** Percentage of the population living below the poverty line; higher values indicate greater socioeconomic disadvantage.
- **Pct_uninsured (%)**: Percentage of individuals without health insurance coverage; higher values suggest greater vulnerability.
- **Pct_renter (%)**: Percentage of households that are renters; higher values reflect a lower rate of homeownership.
- **Pct_crowd (%)**: Percentage of households with more than one occupant per room; higher values indicate greater overcrowding.

*Environmental Exposure covariates include:

- **MeanPM2.5 (µg/m³)**: Average fine particulate matter (PM_2.5_) concentrations (µg/m³) over the study period.
- **MeanNO2 (ppb)**: Average nitrogen dioxide concentrations (ppb) over the study period.
- **MeanO3 (ppb)**: Average ozone concentrations (ppb) over the study period.
- **MeanHumid (g/kg)**: Average specific humidity (g/kg) over the study period.
- **MeanTemp (°C):** Average temperature (Celsius) over the study period.

*Chronic Health Outcome covariates include:

- **Pct_a_disability (%)**: Prevalence of having at least one disability among hearing, vision, cognitive, ambulatory, self-care, or independent living difficulty
- **Stroke (%)**: Prevalence of stroke in the population.
- **Depression (%)**: Prevalence of depression in the population.
- **Diabetes (%)**: Prevalence of diabetes in the population.
- **Obesity (%)**: Prevalence of obesity in the population.
- **Asthma (%)**: Prevalence of asthma in the population.
- **Chronic Obstructive Pulmonary Disease (COPD) (%)**: Prevalence of chronic obstructive pulmonary disease in the population.
- **High Blood Pressure (Hypertension) BPHIGH (%)**: Prevalence of high blood pressure in the population

**Table S2**. State-level estimates of influenza-attributable respiratory emergency department (ED) visit rates per 100,000 population for California (CA), Georgia (GA), and New York (NY); the pooled estimate across all three states was obtained using a random-effects meta-analysis. Values include the point estimate, standard error (SE), and 95% confidence interval (CI).

| **State** | | **Attributable Rate** | **Attributable SE** | **95% CI lower bound** | **95% CI upper bound** | **ICD-coded Influenza Rates** |
| --- | --- | --- | --- | --- | --- | --- |
| **CA** | 232 | | 14 | 206 | 259 | 125 |
| **GA** | 547 | | 21 | 506 | 489 | 405 |
| **NY** | 226 | | 10 | 206 | 245 | 140 |
| **Overall** | 410 | | 16 | 379 | 440 | - |

*All estimates reflect the number of respiratory ED visits per 100,000 population

**Table S3**. Moran’s I statistics for spatial autocorrelation of influenza-attributable respiratory (RESP) Emergency Department (ED) visit rates per 100,000 population across study states.

|  | **Moran I statistic** | **P-value** |
| --- | --- | --- |
| **CA** | 0.1969 | 0.0048 |
| **GA** | 0.1612 | < 0.001 |
| **NY** | 0.0612 | 0.1846 |
| **Overall** | 0.4746 | < 0.001 |

**Table S4.** Spatial meta-regression estimates of county-level poverty and uninsured prevalence in relation to influenza-attributable respiratory emergency department (ED) visit rates per 100,000 population. Models include two-SES adjusted specifications with and without population adjustment in natural spline with three degrees of freedom. Point estimates and 95% credible intervals were obtained using Bayesian MCMC posterior distributions.

| **Covariates** | **Model_4_: No Population Adjusted** | | **Model_4_: Population Adjusted** | |
| --- | --- | --- | --- | --- |
|  | **% Poverty (**$\boldsymbol{\times10\%}$**)** | **% Uninsured (**$\boldsymbol{\times10\%}$**)** | **% Poverty (**$\boldsymbol{\times10\%)}$ | **% Uninsured (**$\boldsymbol{\times10\%)}$ |
| ***Environmental Exposures*** |  |  |  |  |
| PM_2.5_ | 232 (146, 319) | 303 (207, 399) | 225 (142, 310) | 309 (215, 401) |
| NO_2_ | 240 (157, 323) | 296 (199, 393) | 228 (145, 311) | 303 (210, 397) |
| Ozone | 221 (140, 303) | 278 (175, 383) | 214 (136, 294) | 310 (208, 414) |
| Humidity | 192 (113, 272) | 159 (52, 268) | 190 (114, 267) | 154 (38, 263) |
| Temperature | 221 (140, 303) | 251 (139, 366) | 211 (133, 291) | 234 (121, 347) |
| ***Chronic Health Outcome*** |  |  |  |  |
| % Having a Disability | 217 (128, 308) | 365 (244, 480) | 214 (130, 298) | 306 (211, 402) |
| % Stroke | 141 (39, 245) | 257 (157, 361) | 163 (60, 267) | 278 (179, 379) |
| % Depression | 218 (136, 302) | 298 (201, 396) | 222 (143, 302) | 313 (220, 407) |
| % Diabetes | 123 (16, 232) | 226 (118, 335) | 133 (27, 238) | 241 (134, 348) |
| % Obesity | 164 (78, 252) | 284 (191, 379) | 178 (90, 264) | 295 (200, 389) |
| % Asthma | 207 (123, 297) | 296 (200, 393) | 218 (135, 304) | 307 (211, 403) |
| % COPD | 160 (71, 251) | 269 (174, 366) | 170 (78, 262) | 283 (188, 379) |
| % BPHigh | 136 (48, 223) | 240 (146, 334) | 148 (62, 235) | 254 (158, 350) |

**Table S5** Results from spatial meta-regression models examining county-level covariates associated with influenza-attributable respiratory emergency department (ED) visit rates per 100,000 population. Estimates are presented for both the overall annual average and the 2017–2018 peak influenza season. Model 1 includes univariate associations; Model 2 adjusts for poverty rates; Model 3 adjusts for uninsured rates; Model 4 includes both poverty and uninsured rates. **All models additionally account for population size using a cubic spline**. Point estimates and 95% credible intervals are derived from Bayesian MCMC posterior distributions. Estimates and 95% credible intervals were obtained using Bayesian MCMC.

| **Covariates** | **Overall Annual** | | | | **Peak Influenza Season (2017-2018)** | | | |
| --- | --- | --- | --- | --- | --- | --- | --- | --- |
|  | **Model 1** | **Model 2** | **Model 3** | **Model 4** | **Model 1** | **Model 2** | **Model 3** | **Model 4** |
| ***Socioeconomic Status (SES)*** | | | | | | | | |
| % Poverty ( $\times10\%$) | 148 (116, 183) | **-** | 214 (135, 293) | **-** | 374 (301, 449) | **-** | 214 (135, 293) | **-** |
| % Uninsured ( $\times$10%) | 216 (165, 266) | 305 (212, 400) | - |  | 465 (383, 548) | 306 (212, 400) | - |  |
| ***Environmental Exposures*** | | | | | | | | |
| PM_2.5_ ( $\times IQR \mu g/m^{3}$) | 7 (-2, 17) | -4 (-22, 15) | 7 (-11, 25) | -12 (-23, -2) | 36 (19, 54) | -4 (-22, 15) | 7 (-11, 25) | -8 (-26, 11) |
| NO_2_ ( $\times IQR ppb$) | -12 (-36, 12) | -43 (-90, 2) | -13 (-56, 30) | -42 (-65, -20) | 26 (-16, 69) | -40 (-84, 5) | -13 (-56, 30) | -34 (-76, 7) |
| Ozone ( $\times IQR ppb$) | 8 (1, 16) | 13 (2, 14) | -2 (-15, 11) | -1 (-10, 8) | 28 (17, 39) | 13 (2, 24) | -2 (14, 11) | 1 (-13, 11) |
| Humidity ( $\times IQR g/kg$) | 219 (178, 258) | 283 (209, 358) | 240 (149, 334) | 108 (61, 155) | 411 (341, 481) | 284 (209, 358) | 240 (150, 334) | 211 (123, 300) |
| Temperature ( $\times IQR ℃$) | 67 (34, 112) | 122 (74, 169) | 67 (12, 124) | -4 (-29, 23) | 206 (154, 260) | 122 (77, 169) | 67 (11, 124) | 61 (9, 113) |
| ***Chronic Health Outcome*** | | | | | | | | |
| % Having a Disability ( $\times10\%$) | 86 (32,143) | 38 (-70, 146) | 90 (-15, 196) | 15 (-41, 72) | 285 (176, 398) | 38 (-70, 146) | 90 (-14, 195) | 0 (-102, 105) |
| % Stroke ( $\times10\%$) | 1563 (1262, 1859) | 1380 (628, 2132) | 1411 (904, 2018) | 824 (336, 1306) | 2616 (2153, 3072) | 1388 (638, 2127) | 1411 (792, 2022) | 619 (-181, 1397) |
| % Depression ( $\times10\%$) | 3 (-58, 64) | -2 (-9, 9) | -14 (-107, 78) | -54 (-116, 8) | 161 (71, 248) | -3 (-95, 89) | -15 (-110, 78) | -55 (-146, 39) |
| % Diabetes ( $\times10\%$) | 458 (370, 550) | 633 (392, 869) | 556 (346, 760) | 225 (65, 383) | 885 (736, 1033) | 635 (393, 872) | 556 (345, 766) | 321 (44, 598) |
| % Obesity ( $\times10\%$) | 126 (93, 160) | 82 (21, 143) | 109 (54, 163) | 75 (44, 107) | 223 (166, 281) | 81 (23, 141) | 109 (54, 163) | 60 (2, 119) |
| % Asthma ( $\times10\%$) | 265 (120, 412) | 50 (-164, 263) | 150 (-55, 357) | 29 (-131, 187) | 508 (312, 710) | 49 (-165, 263) | 151 (-51, 353) | -24 (-248, 187) |
| % COPD ( $\times10\%$) | 725 (564, 891) | 530 (216, 844) | 590 (321, 863) | 403 (239, 567) | 1174 (925, 1427) | 530 (216, 847) | 579 (310, 847) | 200 (-2, 603) |
| % BPHigh ( $\times10\%$) | 229 (193, 264) | 192 (114, 266) | 188 (118, 256) | 156 (115, 197) | 325 (265, 385) | 191 (116, 266) | 203 (125, 295) | 132 (54, 209) |

**Table S6.** Results of spatial meta-regression on county-level covariates associated with peak influenza season (2017-18)’s influenza-attributable respiratory emergency department (ED) visit rates per 100,000 population. Model 1 includes univariate associations; Model 2 adjusts for poverty rates; Model 3 adjusts for uninsured rates; Model 4 includes both poverty and uninsured rates as covariates. Point estimates and 95% credible intervals are derived from Bayesian MCMC posterior distribution.

| **Covariates** | **Model_1_** | **Model_2_** | **Model_3_** | **Model_4_** |
| --- | --- | --- | --- | --- |
| ***Socioeconomic Status (SES)*** |  |  |  |  |
| % Poverty ( $\times10\%$) | 381 (309, 458) | - | 222 (139, 304) | - |
| % Uninsured ( $\times$10%) | 474 (389, 559) | 299 (203, 397) | - |  |
| ***Environmental Exposures*** |  |  |  |  |
| PM_2.5_ ( $\times IQR \mu g/m^{3}$) | 34 (18, 50) | 4 (-20, 13) | 5 (-8, 24) | -6 (-22, 11) |
| NO_2_ ( $\times IQR ppb$) | -1 (-32, 31) | 43 (-74, 113) | -27 (-57, 4) | -39 (-69, -10) |
| Ozone ( $\times IQR ppb$) | 31 (21, 41) | 17 (6, 27) | 7 (-4, 18) | 7 (-5, 18) |
| Humidity ( $\times IQR g/\mathrm{kg}$) | 403 (334, 470) | 269 (197, 342) | 237 (152, 325) | 204 (121, 286) |
| Temperature ( $\times IQR ℃$) | 182 (130, 238) | 100 (56, 146) | 50 (-3, 104) | 43 (-6, 93) |
| ***Chronic Health Outcome*** |  |  |  |  |
| % Having a Disability ( $\times10\%$) | 295 (195, 398) | 83 (-15, 182) | 128 (35, 222) | 55 (-38, 150) |
| % Stroke ( $\times10\%$) | 2614 (2143, 3075) | 1527 (806, 2247) | 1551 (974, 2119) | 925 (187, 1662) |
| % Depression ( $\times10\%$) | 189 (112, 269) | 54 (-28, 135) | 58 (-22, 138) | 23 (-58, 103) |
| % Diabetes ( $\times10\%$) | 880 (736, 1023) | 637 (403, 873) | 578 (376, 777) | 376 (104, 642) |
| % Obesity ( $\times10\%$) | 224 (171, 278) | 100 (45, 156) | 127 (77, 176) | 87 (35, 138) |
| % Asthma ( $\times10\%$) | 548 (373, 723) | 152 (-40, 343) | 250 (64, 432) | 105 (-91, 30) |
| % COPD ( $\times10\%$) | 1089 (856, 1320) | 545 (283, 807) | 708 (462, 921) | 392 (141, 642) |
| % BPHigh ( $\times10\%$) | 320 (263, 376) | 199 (126, 269) | 202 (137, 266) | 153 (83, 225) |

**Table S7.** Estimated differences in RESP ED visit rates across meteorological categories defined by temperature and humidity levels. Values for “Medium” and “High” represent estimated differences compared with the reference group (Low), not absolute visit rates.

| **Effect Modifier** | **Temperature** | **Humidity** |
| --- | --- | --- |
| **Low: <33^rd^; High: > 66^th^** |  |  |
| Medium (33^rd^ – 66^th^) vs. Low (< 33^rd^) | 79.9 (26.5, 146.8) | 74.7 (29.8, 124.6) |
| High (> 66^th^) vs. Low (<33^rd^) | 197.6 (133.3, 269.3) | 323.7 (262.4, 383.7) |
| **Low: <10^th^; High: >90^th^** |  |  |
| Medium (10^rd^ – 90^th^) vs. Low (< 10^rd^) | 14.8 (-19.3, 50.3) | 21.9 (-12.7, 58.1) |
| High (> 90^th^) vs. Low (<10^rd^) | 145.0 (72.5, 221.7) | 210.7 (180.3, 241.2) |

Note:

| Quantile | 10^th^ | 33^rd^ | 66^th^ | 90^th^ |
| --- | --- | --- | --- | --- |
| Temperature ($℃$) | 2.6 | 11.1 | 13.7 | 15.5 |
| Humidity ($g/\mathrm{kg}$) | 4.3 | 5.8 | 7.6 | 8.9 |

**Figure S1**. Heatmap of season specific estimated influenza-attributable respiratory emergency department (ED) visit rates per 100,000 population for California (CA, 2005-06 to 2017-18), Georgia (GA, 2010-11 to 2017-18), and New York (NY, 2005-06 to 2017-18).

| 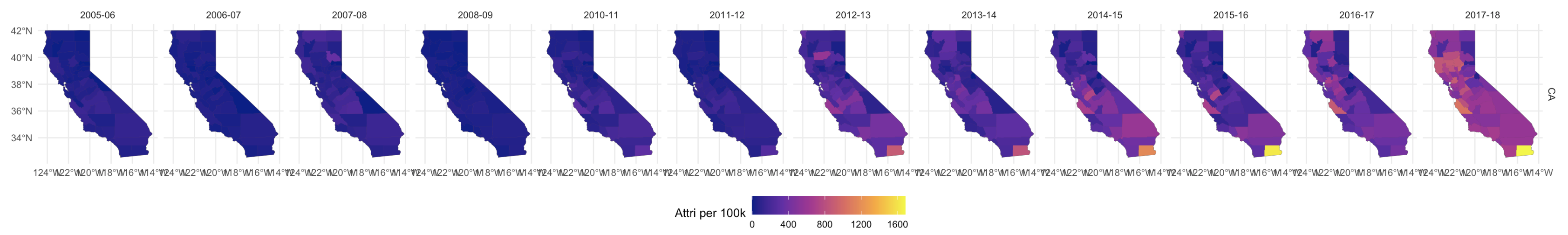 |
| --- |
| 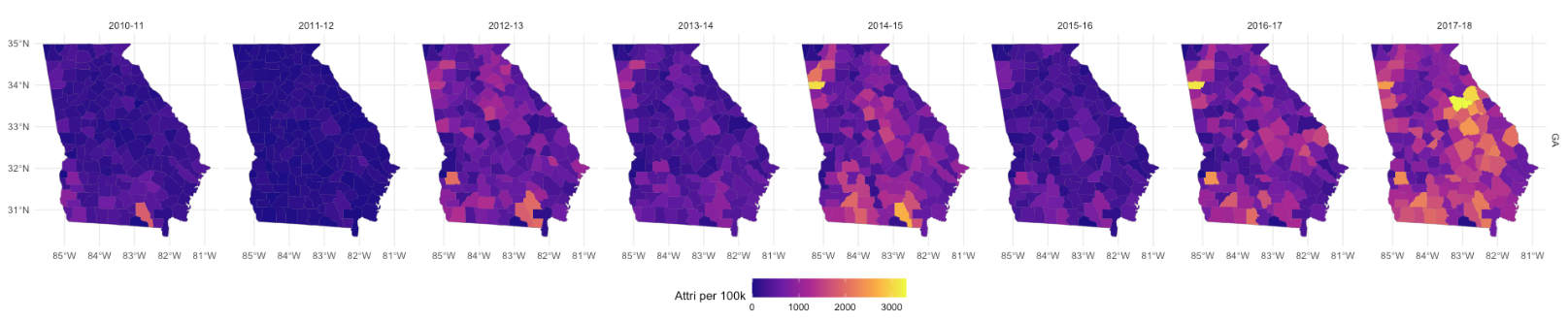 |
| 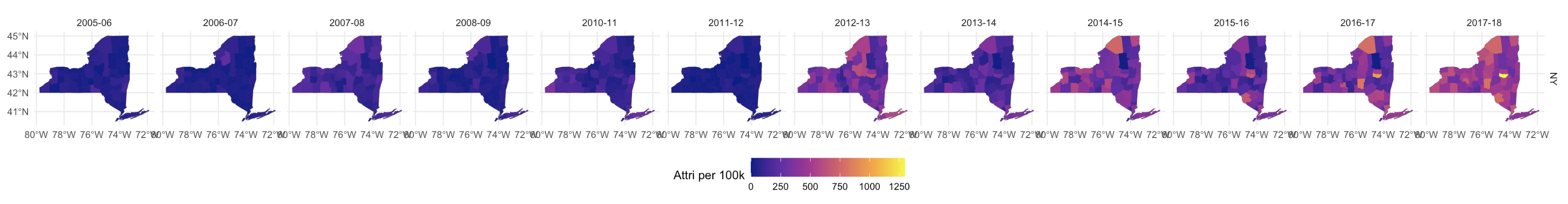 |

**Figure S2**. Spline plot showing the relationship between logarithm of county population and influenza-attributable respiratory ED visit rates per 100,000 population for CA, GA, and NY using a population only spatial meta-regression model.


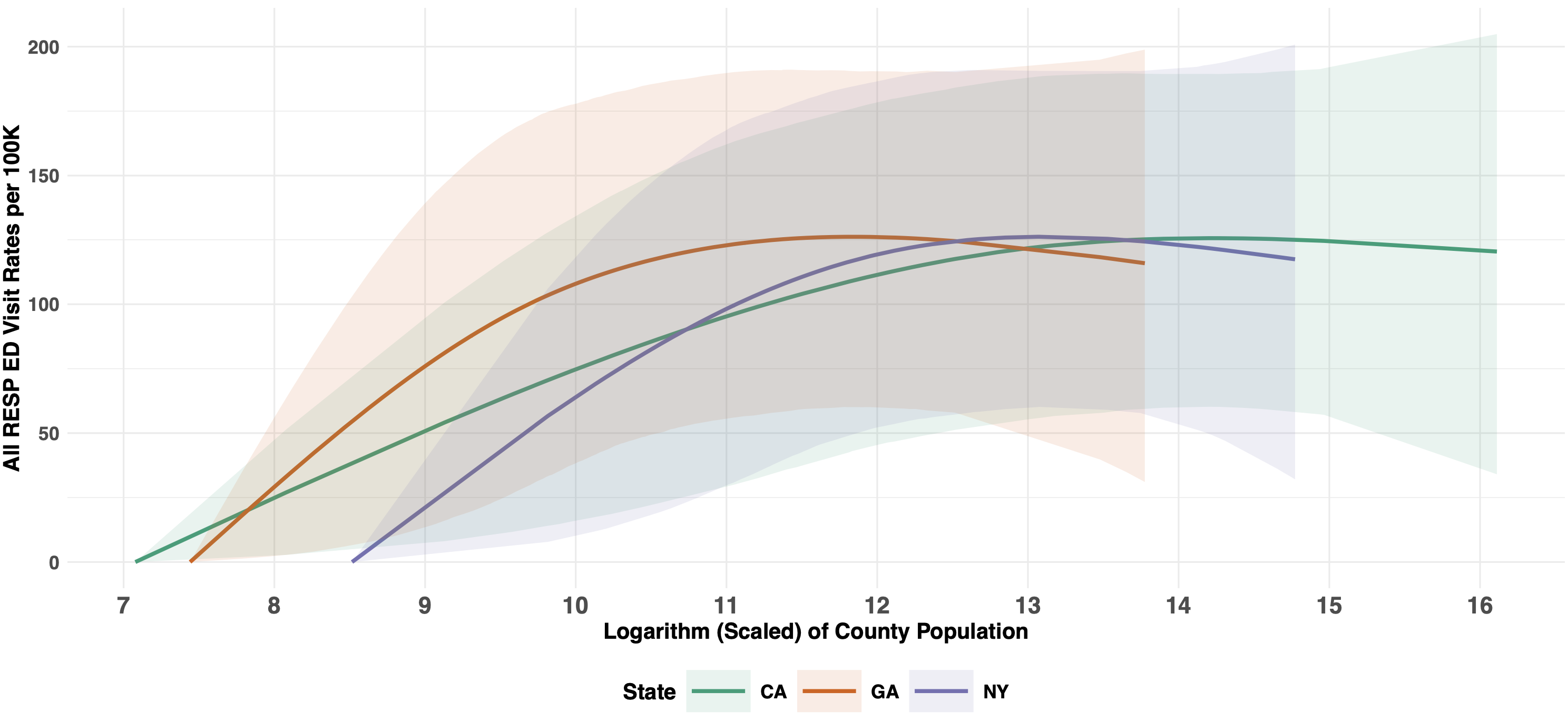


| ns(Log POP, df = 3) | Univariate Model |
| --- | --- |
| ns 1 | 100 (36, 165) |
| ns 2 | 204 (74, 335) |
| ns 3 | 72 (8, 134) |

**Figure S3**. Scatter plot with Pearson Correlation results between laboratory-confirmed influenza counts from FluSurv-NET and hospital discharge influenza counts (ICD-coded) within the same county.


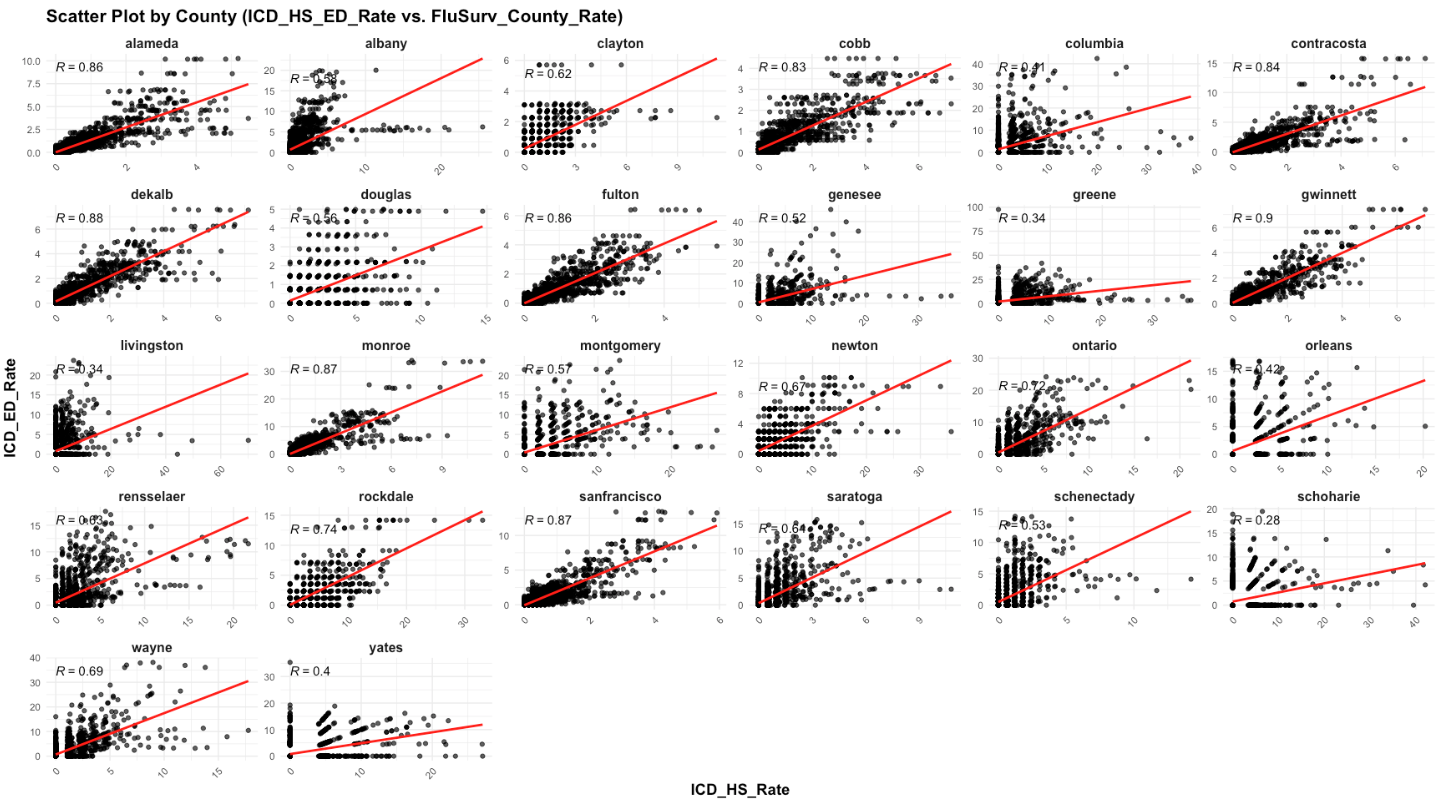

Supplement: Multimedia Appendix 1 [file publichealth_v11i1e82879_app1.docx]
