# Supplementary material for: County-Level Influenza-Attributable Emergency Department Visits and Their Spatial Correlates in the United States: Cross-Sectional Observational Study
Source: JMIR Public Health Surveill. 2025 Dec 30;11:e82879. doi: 10.2196/82879 (PMC12800733; doi:10.2196/82879)
Supplement: Multimedia Appendix 2 [file publichealth_v11i1e82879_app2.docx]

**Statistical Model**

$$\hat{\theta_{ij}}\mid\theta_{ij}\sim^{\text{ind}}\mathcal{N}\left( \theta_{ij},\delta_{ij}^{2} \right),\quad i=1,\ldots,n;\quad j=1,\ldots,m_{i};$$

- $n$: Number of geographically separated spatial regions (e.g., states);
- $m_{i}$: Number of contiguous spatial units within spatial region $i$ (e.g., counties within a state);
- $\hat{\theta_{ij}}$: Point estimate from first stage modeling;
- $\delta_{ij}$: Standard error of the point estimate.

**Stage 2: Bayesian Spatial Hierarchical Modeling**

We used spatial meta-regression to examine associations between county-level RESP ED visits rates and spatial covariates while accounting for spatial autocorrelation. The true county-level burden rate $\theta_{ij}$ is modeled as:

$\theta_{ij}=x_{ij}^{T}\beta+\phi_{ij}+\epsilon_{ij},$ $\epsilon_{ij}\sim N\left( 0,\sigma_{\epsilon}^{2} \right)$

where $x_{ij}^{T}\beta$ represents the fixed effects of the covariates for county $j$ in state $i$. $x_{ij}$ is a vector of county-level covariates with corresponding regression coefficient $\beta$. $\phi_{ij}$ is a random spatial effect that captures residual spatially dependent heterogeneity among counties in each state

This hierarchical model leads to the following conditional distributions:

**Level 1:**

$$\theta_{i} | \beta,\phi_{i},\sigma_{i}^{2} \sim MVN(X_{i}\beta+\phi_{i},\sigma_{i}^{2}I_{m_{i}}),$$

where $X_{i}$ is the $m_{i}\times p$ design matrix for state $i$, and $I_{m_{i}}$ is the identity matrix of size $m_{i}$, the number of counties in state $i$.

**Level 2:**

$$\phi_{i} | \tau_{i}^{2},\rho_{i} \sim MVN(01_{m_{i}},\tau_{i}^{2}Q_{i}\left( \rho_{i} \right)^{-1}),$$

where the $\tau_{i}^{2}$ is the spatial variance parameter controlling the magnitude of spatial variation. $\rho_{i}$ is a spatial correlation parameter. $Q_{i}\left( \rho_{i} \right)^{-1}$ is the precision matrix derived from the adjacency structure $W_{i}$, capturing the spatial relationships among counties within state $i$. These relationships are defined using a basic binary coding approach, where an adjacency matrix assigns a value of 1 if two counties share a border and 0 otherwise.

The priors for this hierarchical model are:

$$\beta\sim N\left( 0,{100}^{2} \right),\tau_{i}^{2}\sim IG\left( 0.01,0.01 \right),\rho_{i} \sim Uniform(0,1),\sigma_{\epsilon}^{2} \sim IG(0.01, 0.01)$$

Inference was conducted using Markov Chain Monte Carlo (MCMC) simulations, implemented to sample from the full conditional distributions of county-level RESP ED visit rates attributable to influenza. For each state and its counties, posterior distributions of  $\theta_{ij}$and $\beta$ were summarized using the posterior means and 95% credible intervals (CrIs). The MCMC algorithm was executed for 20,000 iterations, with the first 2,000 iterations discarded as burn-in to ensure convergence. Diagnostic checks confirmed model stability and reliable parameter estimation.
